# Genome-wide analysis of *PYL-PP2C-SnRK2s* family in *Camellia sinensis*

**DOI:** 10.1080/21655979.2019.1710932

**Published:** 2020-01-11

**Authors:** Ping Xu, Xueying Zhang, Hui Su, Xiaofen Liu, Yuefei Wang, Gaojie Hong

**Affiliations:** a Department of Tea Science, Zhejiang University, Hangzhou, China; b State Key Laboratory for Managing Biotic and Chemical Threats to the Quality and Safety of Agro-products, Institute of Virology and Biotechnology, Zhejiang Academy of Agricultural Sciences, Hangzhou, China; c National Engineering Laboratory of Cold Chain Logistics Technology and Facility for Horticultural Produce, Zhejiang University, Hangzhou, China

**Keywords:** Abscisic acid, *PYLs*, *PP2Cs*, *SnRK2*, *Camellia sinensis*, gene regulatory network

## Abstract

Abscisic acid (ABA) signaling regulates plant growth and development and participates in response to abiotic stressors. However, details about the *PYL-PP2C-SnRK2* gene family, which is the core component of ABA signaling in *Camellia sinensis*, are unknown. In this work, we identified 14 pyrabactin resistance-likes (*PYLs*), 84 type 2C protein phosphatase (*PP2Cs*), and 8 SNF1-related protein kinase 2s (*SnRK2s*) from *C. sinensis*. The transcriptomic analysis indicated that *PYL-PP2C-SnRK2s* were associated with changes of leaf color and the response of *C. sinensis* to drought and salt stressors. Changes of the expression of *Snrk2s* were not significant in the process of leaf color change or drought and salt stress response, suggesting that *PYLs* and *PP2Cs* may not interact with *SnRK2s* in *C. sinensis* during these processes. Finally, Gene Regulatory Network (GRN) construction and interaction networks analysis demonstrated that *PYLs* and *PP2Cs* were associated with multiple metabolic pathways during the changes of leaf color.

## Graphical Abstract

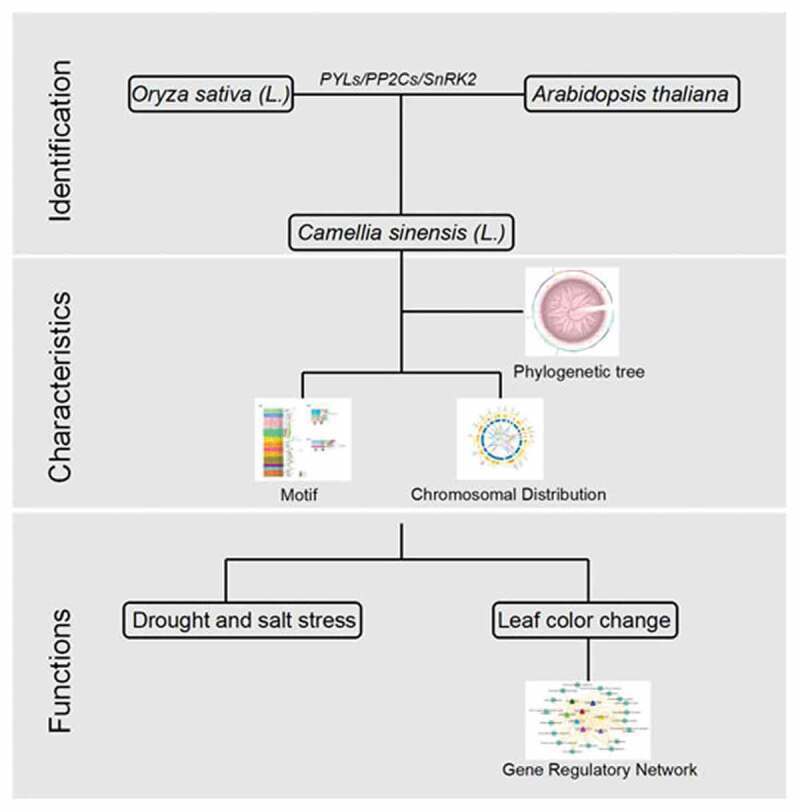

## 1. Introduction

Abscisic acid (ABA) is a phytohormone that plays an important role in plant growth and development including seed germination and fruit ripening [1–5]. In addition, ABA signaling pathways improve plant tolerance to multiple abiotic stressors, such as drought, salinity, and cold [6].

Although numerous factors associated with ABA responses had been identified prior to 2009 [7], the model of ABA signaling pathway is limited. Multiple components of the ABA signaling pathway, such as the ABA receptors and binding proteins, were randomly located in different cellular locations [8]. Since 2009, the discovery of *PYL-PP2C-SnRK2s* as a key component to ABA responses has allowed for a more in depth understanding of ABA signaling. In the *PYL-PP2C-SnRK2* gene family, type 2C protein phosphatase (PP2C) and SNF1-related protein kinase 2 (SnRK2) are downstream components of a type of soluble ABA receptor, *PYR* (pyrabactin resistance)_*/PYL* (PYR1-like)*/RCARs* (regulatory components of ABA receptor) [9–12]. ABA signaling as a double negative regulatory model consists of four stages, including *PYR/PYL/RCARs, PP2Cs, SnRK2s*, and their downstream targets [11,13]. In the absence of ABA, Group A *PP2Cs* inhibits the activity of *SnRKs* by dephosphorylation [7,14,15]. ABA signaling is activated by three stages in the presence of ABA. First, the change of structure of *PYR/PYL/RCARs* is caused by the interaction between ABA, and receptors and then *PYR/PYL/RCARs* bind *PP2Cs* to repress phosphatase activities of *PP2Cs* [10]. Second, kinase activity of *SnRK2s* released from the *PP2C-SnRK2* complex is restored by self-phosphorylation. In the last stage, *SnRK2s* activated downstream transcription factors, which regulated ABA response genes [11,16,17].

Additionally, the members of the *PYL-PP2C-SnRK2* gene family have been characterized and they are involved in growth, development, and a variety of responses to abiotic stressors. Pyrabactin resistance1 (*PYR1*), an ABA receptor, was first discovered in *Arabidopsis* [10]. Subsequently, multiple *PYLs* were identified in *Arabidopsis*, such as *RCAR1* [9]. All *PYLs* belong to the START/Betvl protein family, which contains a START domain [6]. Overexpression of the *PYL* genes *GhPYL10, GhPYL12*, and *GhPYL26* from *Gossypium* spp. in *Arabidopsis* enhanced the plant tolerance to drought stress [18]. Conversely, the quadruple mutant of *pyr1, pyl1, pyl2*, and *pyl4* in *Arabidopsis* was insensitive to ABA [10]. In rice, *OsPYL2, OsPYL10*, and *OsPYL11*, positive regulators of ABA, play an important role in seed germination, early seedling development, drought tolerance, and cold tolerance [19]. As a core component of ABA signaling, *PP2C* proteins contain a conserved catalytic domain on the C-terminus. In *Arabidopsis*, a total of nine Group A *PP2Cs* were obtained. Of these *PP2Cs, AtABll* and *AtABl2* can regulate the development and stomatal movement of *Arabidopsis* in the late stage of germination [9]. *TaPP2C* genes are related to developmental processes and stress responses in *Triticum aestivum* [20]. Significant ABA hypersensitivity was observed after loss-of-function mutations of Group A *PP2Cs*, such as *ABI1, ABI2, HAB1, HAB2, AHG1*, and *PP2CA* [7,21–25], indicating that they play a negative regulatory role in ABA signaling. As a unique gene family in plants, the *SnRK2* family consists of 10 members in *Arabidopsis*, including *SnRK2.1-SnRK2.10*. In these *SnRKs, AtSnRK2.2, AtSnRK2.3, AtSnRK2.6, AtSnRK2.7*, and *AtSnRK2.8* can be induced by ABA [26,27]. Overexpression of *OsSAPK6* in rice showed high sensitivity to ABA [28].

*Camellia sinensis* (tea), a highly nutritious woody plant, is widely distributed, especially in subtropical to tropical regions [29]. Although, the function of *PYL-PP2C-SnRK2s* in plant development and stress responses is well known, the role of *PYL-PP2C-SnRK2s* in *C. sinensis* has not yet been studied. In this study, we aimed to identify the *PYL-PP2C-SnRK2s* family from the whole genome of *C.sinensis* and investigate the functions of the family in abiotic stress and growth and development. This is the first functional study of the *PYL-PP2C-SnRK2s* family in *C. sinensis* and their phylogenetic relationships, chromosome distribution, protein motifs, gene structure, and expression patterns under drought and salt stress as well as in leaves of different colors were investigated. Further, the gene regulatory network (GRN) between *PYL-PP2C-SnRK2s* (regulators) and their co-expression genes (targets) was constructed. This systematic study will enable us to better understand the role of the core components of ABA signaling in *C. sinensis* and provide a solid foundation for improving the yield and quality of *C. sinensis*.

## 2. Materials and methods

### 2.1. Identification of PYL-PP2C-SnRK2 genes in C. sinensis

Protein sequences of *C. sinensis* var. *sinensis* were downloaded from the previously published genome database (http://tpia.teaplant.org/) [30]. The *Arabidopsis* Information Resource (TAIR) (https://www.arabidopsis.org/download/index.jsp/) and Rice Genome Annotation Project (RGAP) (http://rice.plantbiology.msu.edu/) were used to obtain *PYL, PP2C*, and *SnRK2* protein sequences of *Arabidopsis* and rice, respectively [31]. All *PYL-PP2C-SnRK2s* from *Arabidopsis* and rice were used as queries to identify tea *PYL, PP2C*, and *SnRK2* genes from the *C. sinensis* database by employing HMMER software and BLAST. The conserved domains of candidate *PYL-PP2C-SnRK2s* were verified with the online programs NCBI’s conserved domain database (CCD) and protein families database (PFAM) [32,33]. All *PYL-PP2C-SnRK2s* identified in *C. sinensis* are shown in Table S1. The abbreviation of the species name *Camellia sinensis* (*Cs*) is the beginning of each gene name and the most prominent *Arabidopsis* gene from this subfamily was defined as the followed name. Genes not found in *Arabidopsis* are named after the rice gene. The *PYL-PP2C-SnRK2* proteins from *C. sinensis, Arabidopsis*, and rice were selected to construct a Maximum Likelihood tree using MEGA 7.0 software (bootstrap values for 1,000) [34,35].

### 2.2. Conserved motifs and gene structure analysis of C. sinensis PYL-PP2C-SnRK2s

Motifs of *C. sinensis PYL-PP2C-SnRK2s* were identified by using the online program MEME with the maximum number of motifs set to 10 as the parameter. All motifs identified were annotated using InterProScan [36,37]. The gene structure of *PYL-PP2C-SnRK2s* was analyzed by using the Gene Structure Display Server (GSDS) [38]. The composite picture of gene structure and the phylogenetic tree was generated using Tbtools software [39].

### 2.3. Chromosomal distribution and gene duplication

The R package Rideogram and Tbtools were used to show the distribution of all *C. sinensis PYL-PP2C-SnRK2s* on scaffolds.

### 2.4. Transcriptomic analysis and gene expression patterns

All transcriptomic data were obtained from the publicly available NCBI-SRA database (Drought and salt stressors: ERP012919; different leaf colors: SRP055910). The raw data in SRA format downloaded from the NCBI-SRA database were converted to FASTQ format by using fastq-dump (https://trace.ncbi.nlm.nih.gov/Traces/sra/sra.cgi?view=toolkit_doc&f=fastq-dump). The reference genome was obtained from the previously published genome database CSS(cv.Shuchazao). Subsequently, low quality sequences were removed to generate clean reads by FastQC (http://www.bioinformatics.babraham.ac.uk/projects/fastqc/). Three programs were used to analyze clean reads and to obtain the expression levels of all genes based on Fragments Per Kilobase Million (FPKM), HISAT 0.1.5, StringTie (v2.0.4), and Ballgown (a R package) [40–43]. Differentially expressed genes (Log2 fold change >1 or Log2 fold change <−1; *P*-value < 0.05) in drought and salt stress conditions and in leaves of different colors were identified by performing edgeR (http://www.bioconductor.org/packages/release/bioc/html/edgeR.html) and Ballgown, respectively.

### 2.5. KEGG pathway enrichment analysis

To further understand the biological functions of genes, a Kyoto Encyclopedia of Genes and Genomes (KEGG) pathway analysis was performed. The significantly enriched pathway analysis uses KEGG as a unit to apply hypergeometric tests to find pathways that are significantly enriched in differentially expressed genes compared to the entire genomic background. This was calculated as follows [44]:

$$P=1-\overset{m-1}{\underset{i=0}{\sum }}\frac{\left(\begin{array}{c} M\\ i \end{array}\right)\left(\begin{array}{c} N-M\\ n-i \end{array}\right)}{\left(\begin{array}{c} N\\ n \end{array}\right)}$$

where N is the total number of genes; n is the number of differentially expressed genes in N; m is the number of genes annotated as a particular pathway; M is the number of differentially expressed genes annotated as a particular pathway.

### 2.6. Gene regulatory network construction KEGG enrichment analysis

An unsupervised GRN was constructed by using the R package GENIE3 with the random forest machine learning algorithm. In this GRN, a total of 229,504 edges were generated and the top 20% of edges (rank by edge core) were defined as high confidence edges [45].

## 3. Results

### 3.1. Identification and characteristics of *PYL-PP2C-SnRK2s in C. sinensis*

**Figure 1.**
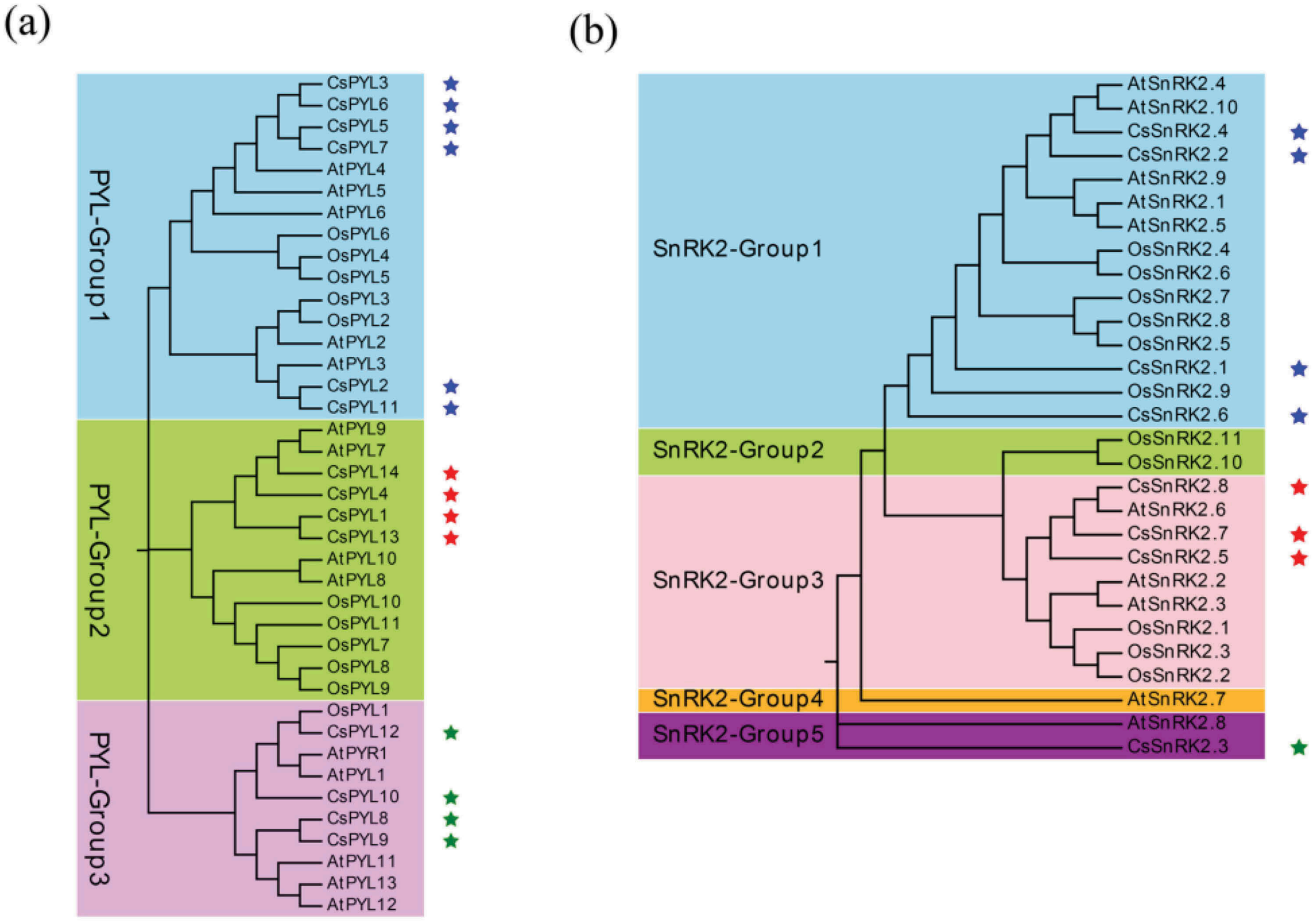
Maximum likelihood phylogeny of PYL (a) and SnRK2 (b) proteins from *C. sinensis, Arabidopsis*, and rice using complete protein sequences. The tree reliability was assessed by using 1,000 bootstrap replicates.

**Figure 2.**
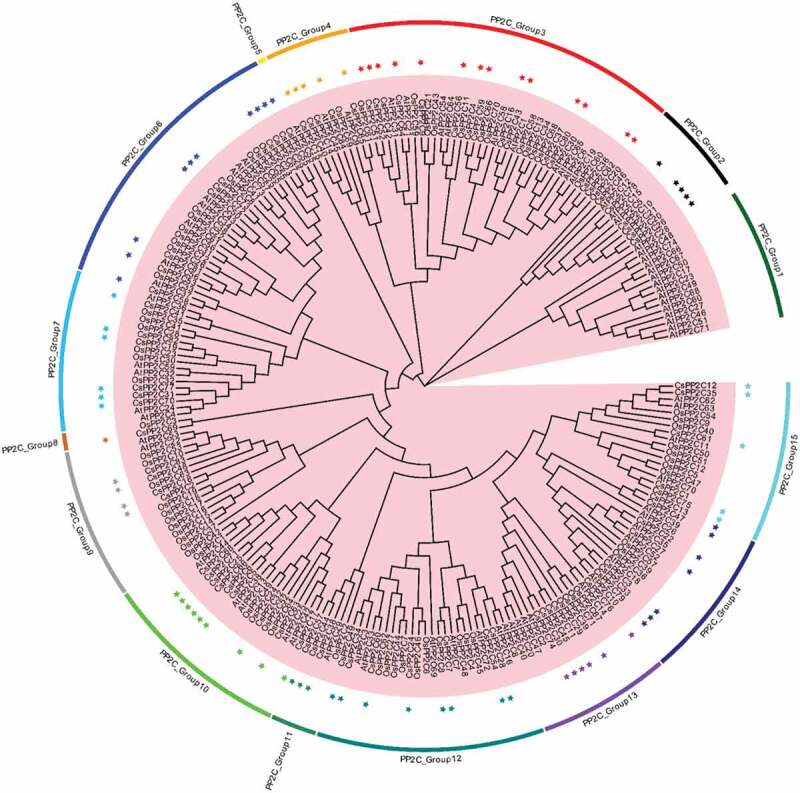
Maximum likelihood phylogeny of PP2C proteins from *C. sinensis, Arabidopsis*, and rice using the complete protein sequences. The tree reliability was assessed by using 1,000 bootstrap replicates.

In total, we identified 8 *SnRK2s*, 84 *PP2C*s, and 14 *PYL*s genes in *C. sinensis* by performing multiple bioinformatics software (Described in materials and methods) (Table S1). Maximum Likelihood phylogenetic trees reconstructed with the complete *PYL-PP2C-SnRK2* protein sequences from *C. sinensis, Arabidopsis*, and rice showed 3 (PYL_Group1-3), 15 (PP2C_Group1-15), and 5 (SnRK2_Group1-5) subgroups of the *PYL, PP2*C, and *SnRK2* families, respectively (Figures 1 and 2). Multiple orthologous *PYL-PP2C-SnRK2s* between *C. sinensis* and *Arabidopsis* were identified, suggesting that relationships with *PYL-PP2C-SnRK2s* between *C. sinensis* and *Arabidopsis* were closer than that between *C. sinensis* and rice. These results suggested that the gene phylogeny was in accordance with the species phylogeny. There was no significant difference in the number of *PYL-PP2C-SnRK2s* in *C. sinensis* (*PYL*: 14, *PP2C*: 84, *SnRK*2: 8), *Arabidopsis* (*PYL*: 14, *PP2C*: 74, *SnRK2*: 10), and rice (*PYL*: 11, *PP2C*: 72, *SnRK2*: 11).

### 3.2. Conserved motifs *and gene structure analysis of C. sinensis P*YL-PP2C-SnRK2s

**Figure 3.**
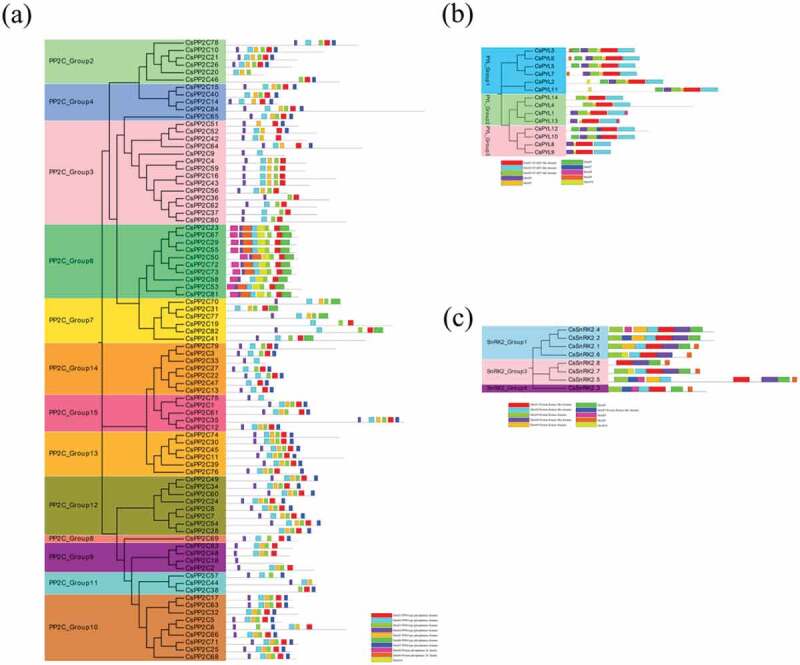
The conserved motifs of *C. sinensis* (a) PP2Cs, (b) PYLs, and (c) SnRK2s based on phylogenetic relationships. All motifs were identified using the MEME database with the complete amino acid sequences of *C. sinensis PP2Cs, PYLs*, and *SnRK2s.*

According to the phylogenetic relationships, the conserved motifs of *C. sinensis PYL-PP2C-SnRK2* proteins were analyzed using MEME and InterPro databases and ten motifs in each gene family were acquired (Figure 3). For the *C. sinensis PP2C* family, the PPM-type phosphatase domain and protein phosphatase 2C families were annotated by protein sequences of *CsPP2Cs* in motifs 1–7 and 8–9, respectively. Motifs 1–4 were included in all of the subgroups and PP2C_Group 6 has motifs 9 and 10, whereas motif 6 was only present in PP2C_Groups 6 and 7 (Figure 3(a)). For the *C. sinensis PYL* family, the protein sequences of motifs 1–3 involved the START-like domain. Motifs 1 and 2 were present in all of the identified *CsPYLs*. Almost all of the *CsPYLs* contained motif 3, except for *CsPYLs* 13, 8, and 9 (Figure 3(b)). In the *C. sinensis SnRK2* family, motifs 1–5 and 7 were annotated as a protein kinase (-like) domain. All the *CsSnRK2s* contained motif 1; motifs 2 and 3 not present in *CsSnRK2.8*, suggesting that all identified *CsSnRK2s* contained protein kinase domains. In addition, the gene structure of *C. sinensis PYLPP2C-SnRK2s* was analyzed; exon-intron organizations in the same subgroups were similar (Figure S1). These results indicate that typical family features exist in all *PYL-PP2C-SnRK2s* obtained from *C. sinensis*.

### 3.3. Chromosomal distribution, gene duplication, and syntenic analysis of C. sinensis PP2C-PYL-SnRK2s

**Figure 4.**
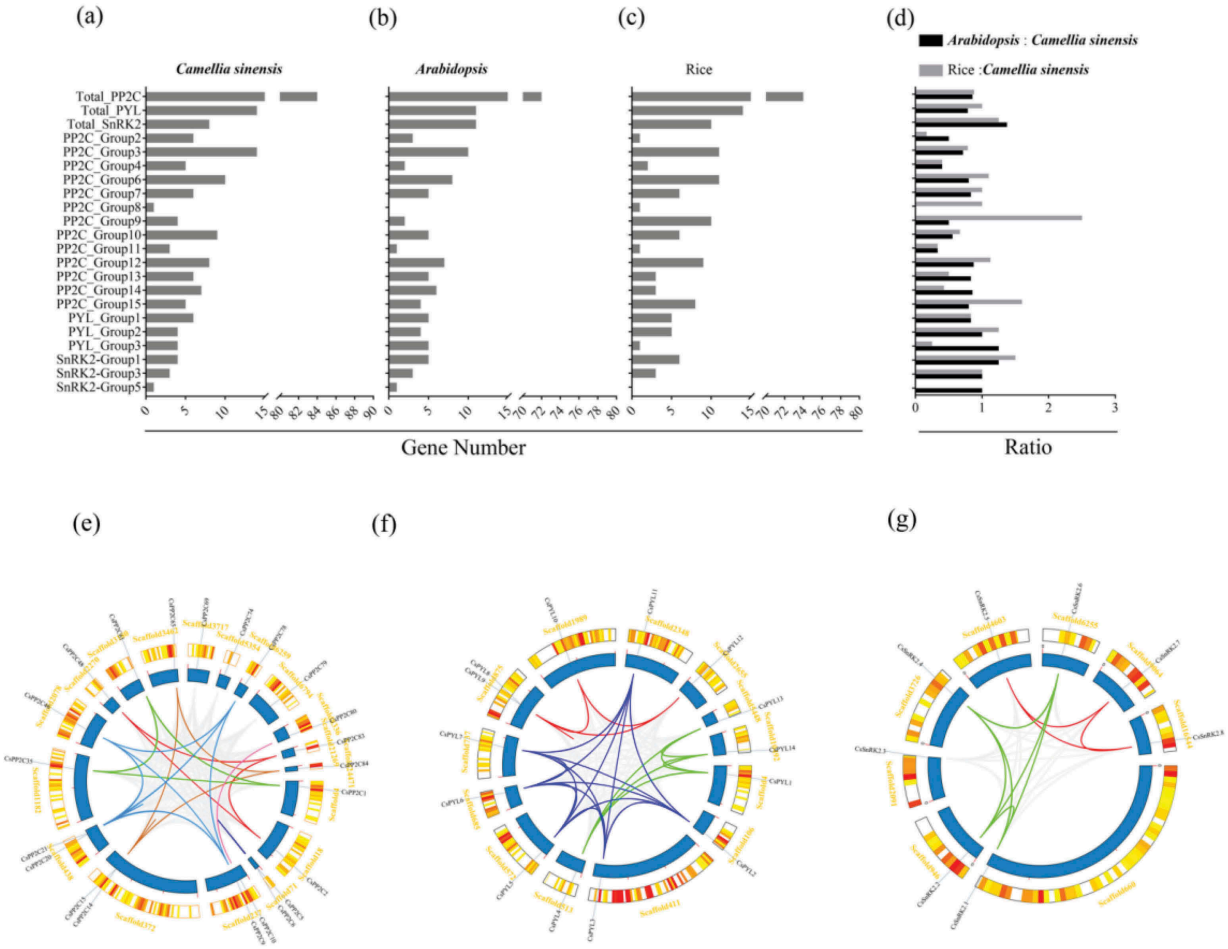
Number and location of PP2C-PYL-SnRK2s. The number of PP2C-PYL-SnRK2s identified per subgroup in (a) *C. sinensis*, (b) *Arabidopsis*, and (c) rice. (d) The ratio of the total number of *PP2C-PYL-SnRK2* genes in all subgroups is shown for *Arabidopsis* compared to *C. sinensis* (black) and rice compared to *C. sinensis* (gray). (e, f, g) All *PP2C-PYL-SnRK2* genes are mapped to the *C. sinensis* scaffolds in a circular diagram using Tbtools. Scaffolds mapped with all PYLs and SnRK2s and scaffolds containing at least 20 PP2C genes are shown. Links represent different genes in the same subgroups.

Scaffolds mapped with *PP2C-PYL-SnRK2s* were shown in the absence of *C. sinensis* chromosome data. There were a total of 95 scaffolds related to all *PP2Cs* (80), *PYLs* (13), and *SnRK2s* (8). Six shared scaffolds were mapped with *PP2C* and *PYL* (Figure S2, Table S2). *PP2C-PYL-SnRK2s* were unevenly distributed on all scaffolds. Despite the phylogenetic distance of rice, *C. sinensis*, and *Arabidopsis*, the number of *PP2C-PYL-SnRK2s* was similar (Figure 4(a–d)). In addition, the number of most orthologs between *C. sinensis* and *Arabidopsis* or rice was similar or even the same in some subgroups, such as PP2C_Group 7 and 8, as well as SnRK2-Group 3 and 5. These results suggest that the number of *PP2C-PYL-SnRK2s* is conserved during evolution. Duplication events were analyzed with Tbtools software to detect syntenic blocks. Syntenic relationships of scaffolds mapped with all *PYLs* and *SnRK2s* and scaffolds containing at least 20 *PP2C* genes were analyzed (Figure 4(e–f)). We found that genes from the same phylogenetic subgroup were located on multiple scaffolds and most gene replication events occurred on the same scaffold.

### 3.4. Expression analysis of PYL-PP2C-SnRK2 genes in response to salt and drought stress

**Figure 5.**
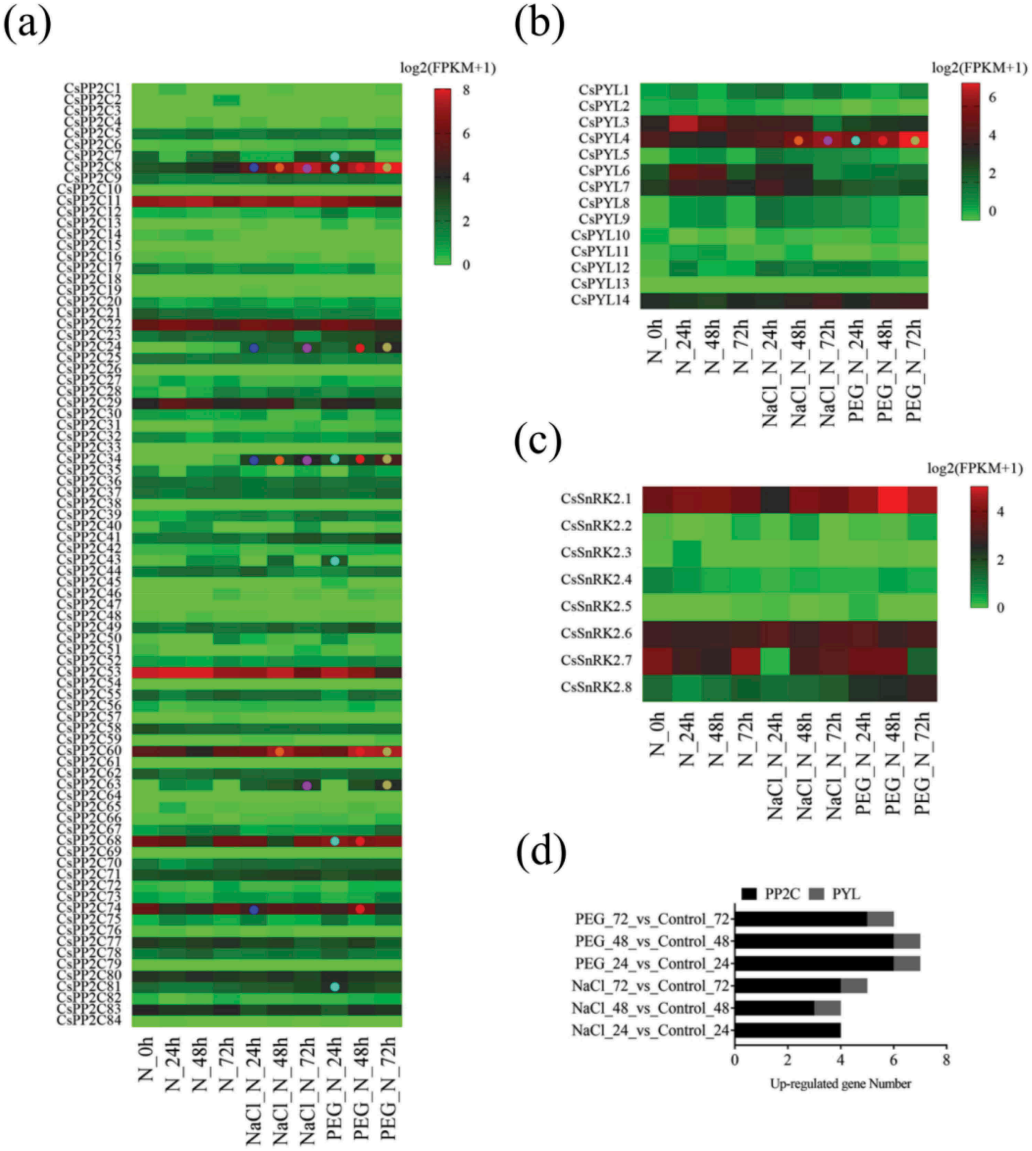
Expression profiles of *PP2Cs* (a), *PYLs* (b), and *SnRK2s* (c) in response to salt and drought treatments in *C. sinensis*. Log2 (FPKM+1) was used to create the heat map. Green indicates low expression, whereas red indicates a high level of expression. Circles of different colors represent differential gene expression during different treatments compared with the controls. (d) The histogram shows the distribution of differential expression of *PP2Cs, PYLs*, and *SnRK2s* in different comparisons.

To explore the role of *PYL-PP2C-SnRK2* genes in *C. sinensis* in response to abiotic stress, transcriptomes of leaves treated with 200 mM NaCl and 25% polyethylene glycol (PEG) for different periods of time (24 h, 48 h, and 72h) were downloaded from the publicly available NCBI-SRA database (ERP012919) (Figure 5, Table S3). Under the NaCl and PEG treatments for 24 h, 0/14 and 1/14 *PYLs*, 4/84 and 6/84 *PP2Cs*, and 0/8 and 0/8 *SnRK2s* showed significant upregulation, respectively (Log2 (fold change) >1; *P*-value < 0.05) (Figure 5 (d)). After 48 h, 1/14 and 1/14 *PYLs*, 3/84 and 6/84 *PP2Cs*, and 0/8 and 0/8 *SnRK2s* showed significant upregulation, respectively. After 72 h, 1/14 and 1/14 *PYLs*, 4/84 and 5/84 *PP2Cs*, and 0/8 and 0/8 *SnRK2s* showed significant upregulation, respectively. Of note, *CsPP2C8* and *CsPP2C24* showed up-regulated expression levels at all treatment stages and *CsPP2C 24, 60, 68, 74* showed high expression levels at multiple stages (Figure 5(a)). In addition, only one *PYL* gene *CsPYL4* was induced by salt and drought stress (Figure 5(b)). These results suggest that *PYL-PP2C-SnRK2* genes were involved in the response of *C. sinensis* to salt and drought stress; overall, six *PP2C* genes (*CsPP2C8, 24, 24, 60, 68*, and *74*) and one *PYL* gene (*CsPYL4*) plays an important role in this process.

### 3.5. Expression analyses of PYL-PP2C-SnRK2 genes during leaf development and color change

**Figure 6.**
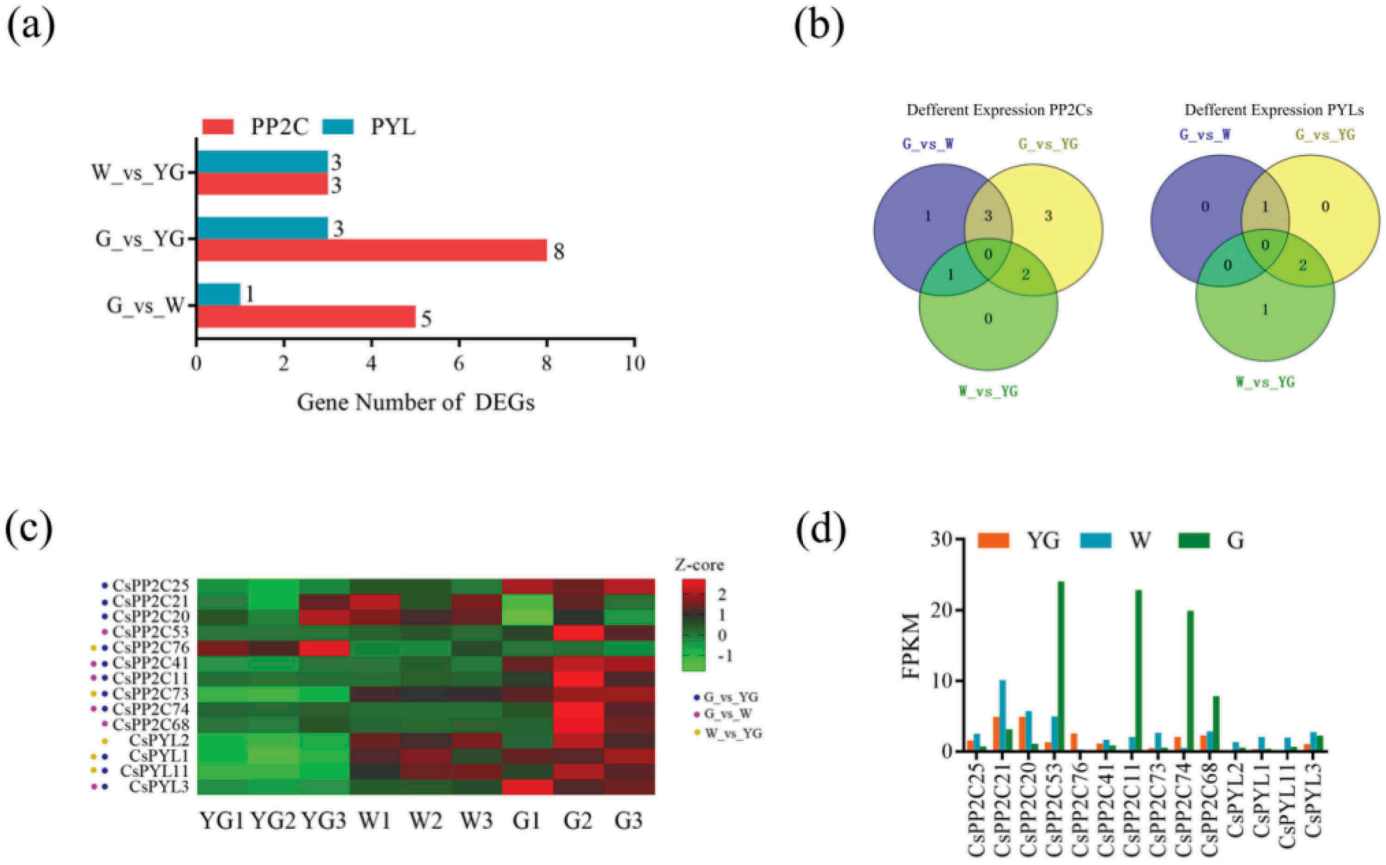
Expression analyses of *PYL-PP2C-SnRK2* genes during leaf development and color change. (a) The histogram shows the number of differential expressed *PYL-PP2C-SnRK2* genes in three comparisons, W_vs_YG, G_vs_YG, and G_vs_W. (b) The Venn diagrams show the distribution of differential expression of *PP2Cs* and *PYLs* in three comparisons. (c) The expression profile of the differential expression of *PP2Cs* and *PYLs* is shown with a heatmap based on the Z-core value. (d) The histogram shows the expression level of all of the differentially expressed *PP2Cs* and *PYLs.*

According to previous report, a total of three stages were defined in the development of new *C. sinensis* shoots, including the yellow-green (YG) stage, albescent (W) stage, and re-greening (G) stage [46]. The changes of leaf color involve a variety of metabolic pathways related to plant growth and development, which was affected by ABA signaling [1–5]. Therefore, *PYL-PP2C-SnRK2* genes may be involved in the changes of color in *C. sinensis* leaves. In order to verify this hypothesis, the transcriptomes of leaves at these three stages were obtained from the publicly available NCBI-SRA database (SRP055910) and expression levels of all mRNAs based on FPKM values was calculated (Figure S3, Table S4). The expression level of all *PYL-PP2C-SnRK2s* from mRNAs was generated (Table S5). In total, 1,437 (up: 839; down: 598) DEGs in G vs W, 3,457 (up: 1,790; down: 1,667) DEGs in G vs YG, and 1,409 (up: 710; down: 699) in W vs YG were generated (Figure S4, Table S6). There were three *PP2Cs* and three *PYLs* in G vs YG, eight *PP2C*s and three *PYLs* in G vs YG, and three *PP2Cs*, and one *PYLs* in G vs W generated from all DEGs (Figure 6(a)). All differentially expressed *PP2Cs* of W vs YG existed in the other two comparisons (Figure 6(b)) and *CsPP2C 11 (CSS038291), 41 (CSS030837), 73 (CSS047922), 74 (CSS049314), 76 (CSS029712)* and *CsPYL1 (CSS036090), 11 (CSS044035), 3 (CSS050443)* were differentially expressed in two comparison groups (Figure 6(c)). Compared with stage YG, 9/14 and 7/14 genes were upregulated in the W and G stages (Figure 6(d)). These results suggest that *PP2Cs* and *PYLs* are involved leaf color changes. In addition, changes in the expression levels of *Snrk2s* were not significant, indicating that *PP2Cs* and *PYLs* may affect the changes of leaf color via a mechanism not involving *SnRK2s*.

### 3.6. Regulatory modules of PYL-PP2C-SnRK2 genes related to changes of C. sinensis leaf color

**Figure 7.**
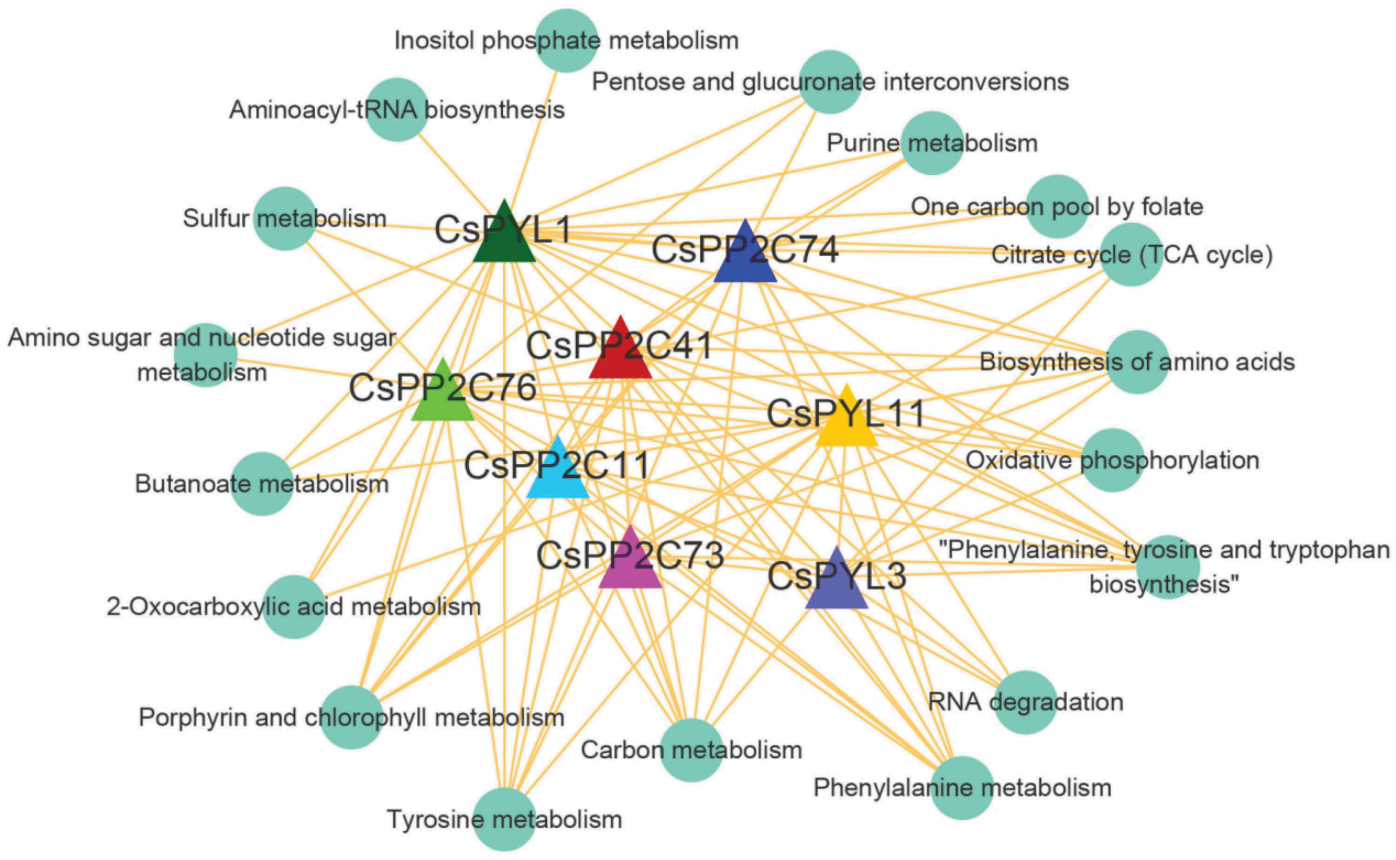
Interaction network between KEGG pathways involving at least 10 target genes and *PYL-PP2C-SnRK2* genes.

To further explore the regulatory patterns of gene expression during changes of leaf color, a GRN was used to create a directed network of *PYL-PP2C-SnRK2* genes and their target genes. Since *CsPP2C 11 (CSS038291), 41 (CSS030837), 73 (CSS047922), 74 (CSS049314), 76 (CSS029712)* and *CsPYL1 (CSS036090), 11 (CSS044035), 3 (CSS050443)* were differentially expressed in two comparison groups, they were used to construct the GRN as regulators and 28,682 co-expression mRNAs as target genes. An independent GRN was generated, which included 229,504 edges and the top 20% edges (rank by edge core) (Table S7). All target genes of each regulator were subjected to a KEGG pathway enrichment analysis. Pathways involving at least ten target genes were used to construct an interaction network with regulators (Figure 7). In this network, four pathways, ‘Carbon metabolism,’ ‘Phenylalanine metabolism,’ ‘Phenylalanine, tyrosine, and tryptophan biosynthesis,’ and ‘Biosynthesis of amino acids’ were affected by all regulators. ‘Tyrosine metabolism’ and ‘Porphyrin and chlorophyll metabolism’ were involved in almost all regulators, except *CsPYL3*. There were at least four regulators associated with the pathways ‘RNA degradation,’ ‘Citrate cycle (TCA cycle),’ and ‘Oxidative phosphorylation.’ In addition, the pathways ‘Aminoacyl-tRNA biosynthesis’ and ‘Inositol phosphate metabolism’ were only regulated by *CsPYL1*. These results indicated that *PYL-PP2C-SnRK2s* may affect the change of *C. sinensis* leaf color by regulating multiple metabolic pathways.

## 4. Discussion

The ABA signaling pathway allows a plant to respond to stress conditions, such as drought, salt, and cold stressors [47–49]. The characteristic of the *PYL-PP2C-SnRK2* gene family, which is a core regulatory network of the ABA pathway in *C. sinensis* is unknown. Here, we identified eight *SnRK2s*, 84 *PP2C*s, and 14 *PYL*s from the *C. sinensis* genome, which were assigned to four, 13, and three conserved subfamilies, respectively, based on a phylogenetic analysis (Figures 1 and 2). The evolutionary relationships of the *PYL-PP2C-SnRK2* gene family between *C. sinensis* and *Arabidopsis* are closer than the relationships between *C. sinensis* and rice, which is consistent with the relatedness of these species. The similarity in the number of *PYL-PP2C-SnRK2* genes in *C. sinensis, Arabidopsis*, and rice indicates conservation. The conserved motif analysis showed that the START-like domain, PPM-type phosphatase domain, and protein kinase domain were contained in all *C. sinensis PYLs, PP2Cs*, and *SnRK2s*, respectively. These characteristics identified in *C. sinensis* are in accordance with other plant species, such as apple and *Arabidopsis* [48,50]. All *PP2C-PYL-SnRK2* genes were unevenly distributed on the scaffolds (Figure S2, TableS2) and replication events of most genes occurred on the same scaffold (Figure 3).

The ABA signaling pathway is related to drought and salt stress response in plants [47,48]. The overexpression of *OsPYL3* and *OsPYL9* genes can increase rice tolerance to drought [51], and *NtPYLs* have been shown to have important functions in the drought tolerance of *Nicotiana tabacum* [52]. High-throughput next generation sequencing showed that *PYL, PP2C*, and *SRK2E* respond to drought stress in *Gossypium* spp [53]. In banana, the *PYL-PP2C-SnRK2* gene family regulated its tolerance to abiotic stress, such as salt and drought stress conditions [54]. In this work, transcriptome data from *C. sinensis* treated with PEG and NaCl were obtained from the NCBI-SRA database and analyzed. For NaCl treatment, there were two *PYLs* and 11 *PP2Cs* that showed significantly upregulation at different stages. A total of three *PYLs* and 17 *PP2Cs* showed up-regulated expression in *C. sinensis* treated PEG. These results suggested that *CsPYLs* and *CsPP2Cs* may be associated with the tolerance of *C. sinensis* to drought and salt stress. In addition, the expression levels of all *CsSnRK2s* were not significant under NaCl and PEG treatments, indicating that *CsSnRK2s* may not be affected by drought and salt stress treatments.

Although, the function of the ABA signaling pathway on plant development is well known, no information is available regarding the effect of ABA on leaf color in *C. sinensis*. Thus, we analyzed the transcriptomes from different colored leaves of *C. sinensis*. In different colored leaves, the expression levels of multiple *PYL-PP2C-SnRKs* were significantly different. For example, *CsPP2C 11 (CSS038291), 41 (CSS030837), 73 (CSS047922), 74 (CSS049314), 76 (CSS029712)* and *CsPYL1 (CSS036090), 11 (CSS044035), 3 (CSS050443)* showed significantly different expression levels, which indicated that *CsPP2Cs* and *CsPYLs* may be involved in the regulation of leaf color changes. In addition, there was no significant change in the expression level of the *CsSnRK2s* in drought, or salt stress treatments, or among different leaf colors, suggesting that compared with *CsSnRK2s, CsPP2Cs* and *CsPYLs* play a more important role in the response of *C. sinensis* to drought and salt stress as well as leaf color.

The relationship between *PYL-PP2C-SnRK2s* and their targets was investigated using GRN, which is a directed network of regulators and their target genes. In this work, the regulators *CsPP2C 11 (CSS038291), 41 (CSS030837), 73 (CSS047922), 74 (CSS049314), 76 (CSS029712)* and *CsPYL1 (CSS036090), 11 (CSS044035), 3 (CSS050443)* were selected to construct a GRN, and 28,682 co-expression genes were selected as target genes. A KEGG enrichment analysis of target genes showed that *CsPP2Cs* or *CsPYLs* are involved in multiple metabolic pathways, such as ‘Phenylalanine metabolism’, ‘Phenylalanine, tyrosine, and tryptophan biosynthesis’, ‘Biosynthesis of amino acids’, and ‘Carbon metabolism’. In plants, phenylalanine metabolism is even more diverse and prevalent pathway. In fact, about 25% of fixed carbon from photosynthesis was used to generate phenylalanine [55] and phenylalanine-derived compounds, which accounts for approximately 40% of plant organic matter [56]. In addition, phenylalanine also affects plant characteristics in many aspects, such as growth and development (lignin) [57], defense (salicylic acid [58], tannins and flavonoids [59]), reproduction (phenylpropanoids and benzenoids) [60] and other metabolic pathways also play an important role in the growth and development of plants, indicating that *CsPP2Cs* and *CsPYLs* are essential for plant survival. *SnRK2s* are important components of the ABA signaling pathway in response to osmotic and salt stress in *Arabidopsis* [61]. Interestingly, the expression levels of all *SnRK2s* did not show significant changes among different leaf colors or under salt and drought treatments in *C. sinensis*. The functional characteristics of *SnRKs* in *C. sinensis* need to be further clarified in future studies. This study improves the understanding of *PYL-PP2C-SnRK2*-mediated ABA signaling in the regulation of leaves change, response to drought and salt stressorse in *C. sinensis*. The identification of some candidate genes provides a molecular basis for improving *C. sinensis* quality and yield.

## 5. Conclusion

In summary, we identified 8 *SnRK2s*, 84 *PP2C*s, and 14 *PYL*s genes from the whole genome of *C. sinensis*. The phylogeny of these genes is in accordance with the species phylogeny. Conserved motifs analysis indicates that all identified *SnRK2s, PP2C*s, and *PYL*s contain protein kinase domain, PPM-type phosphatase domain and START-like domain, respectively. The transcriptomic analysis indicates that The *PYL-PP2C-SnRK2s* are related to *C. sinensis* responses to drought and salt stressors and participate in the regulation mechanism of leaf color change by multiple metabolic pathways. GRN construction and interaction networks analysis demonstrated that PYLs and PP2Cs were associated with multiple metabolic pathways during the changes of leaf color.

## Highlights

1. The *PYL-PP2C-SnRK2* family genes in *Camellia sinensis* were identified.
2. The ABA signaling is related to the response of *C. sinensis* to abiotic stress.
3. The *PYL-PP2C-SnRK2s* play an important role in the change of *C.sinensis* leaf colors.

## Supplementary material

The supplemental data for this article can be accessed here.
