# Supplementary figures and images for: Genome-wide analysis of PYL-PP2C-SnRK2s family in Camellia sinensis
Source: Bioengineered. 2020 Jan 11;11(1):103–15. doi: 10.1080/21655979.2019.1710932 (PMC6961588; doi:10.1080/21655979.2019.1710932)

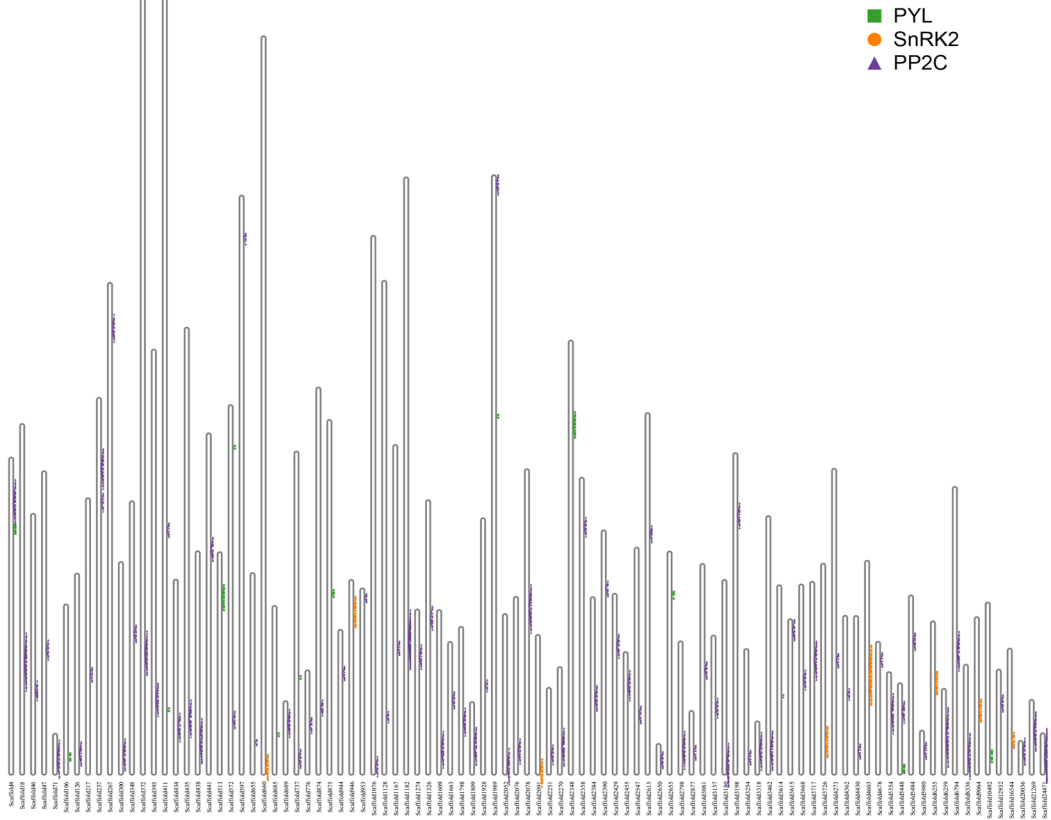

Supplement: Supplemental Material [file kbie-11-01-1710932-s001.zip › Supplementary Files/FigureS2.pdf]

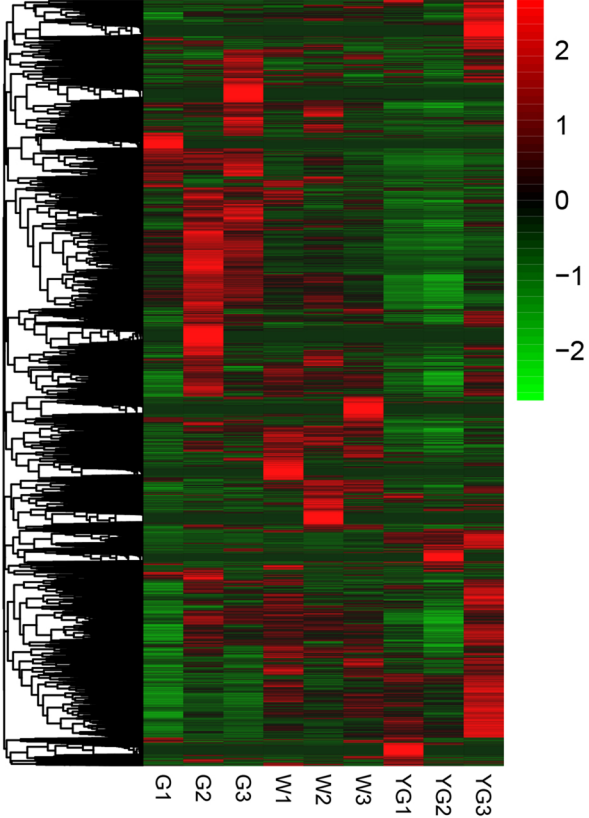

Supplement: Supplemental Material [file kbie-11-01-1710932-s001.zip › Supplementary Files/FigureS3.pdf]

(a)

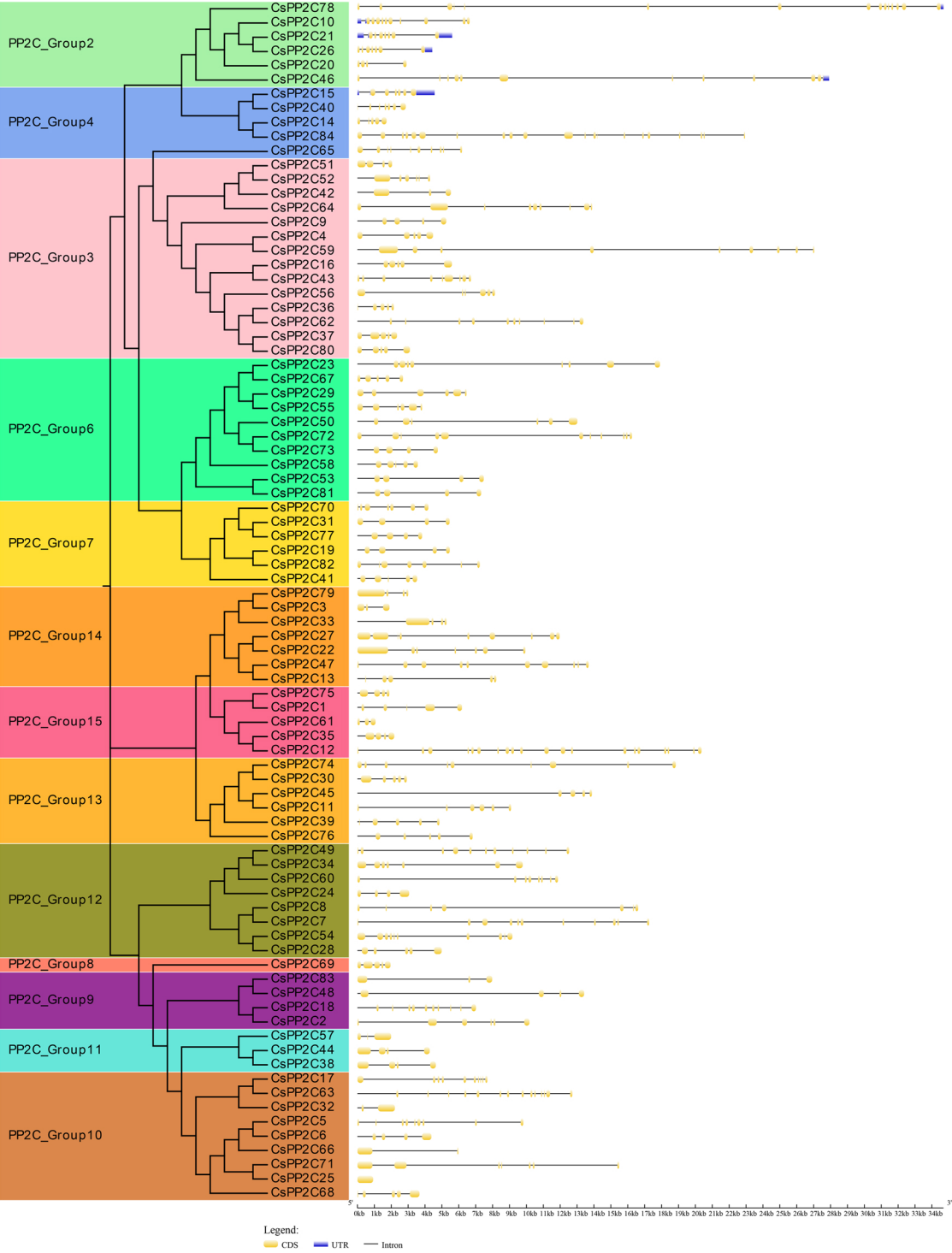

(b)

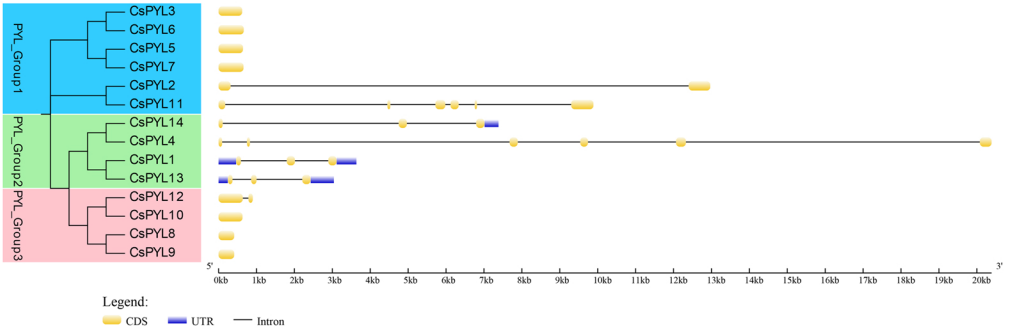

(c)

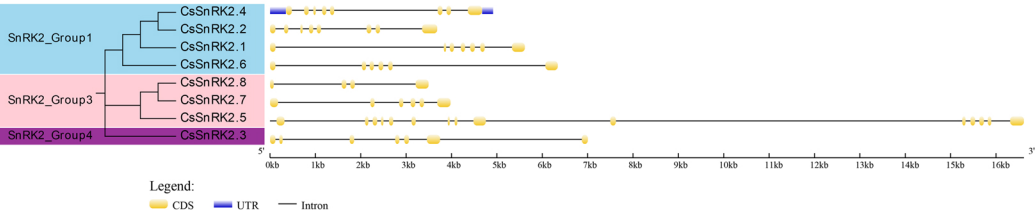

Supplement: Supplemental Material [file kbie-11-01-1710932-s001.zip › Supplementary Files/FigureS1.pdf]

(a)

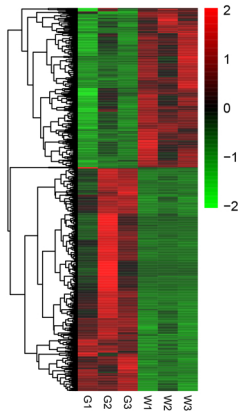

(b)

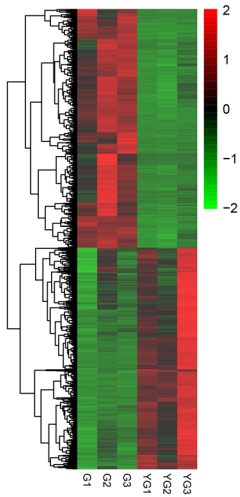

(c)

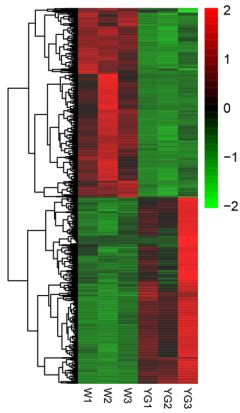

Supplement: Supplemental Material [file kbie-11-01-1710932-s001.zip › Supplementary Files/FigureS4.pdf]
